# The Role of the L-Arginine–Nitric Oxide Molecular Pathway in Autosomal Dominant Polycystic Kidney Disease

**DOI:** 10.3390/jpm14030299

**Published:** 2024-03-11

**Authors:** Corina Daniela Ene, Mircea Penescu, Ilinca Nicolae, Cristina Capusa

**Affiliations:** 1 Faculty of General Medicine, Nephrology and Internal Medicine Department, Carol Davila University of Medicine and Pharmacy, 050474 Bucharest, Romania; 2 Carol Davila Clinical Hospital of Nephrology, 010731 Bucharest, Romania; 3 Research Department, Victor Babes Clinical Hospital of Infectious and Tropical Disease, 030303 Bucharest, Romania

**Keywords:** arginine auxotrophy, autosomal dominant polycystic kidney disease, cystogenesis, inducible nitric oxide synthase, arginase 2, nitric oxide metabolites, dimethylarginine

## Abstract

Recently, arginine has been proven to play an important role in ADPKD physiopathology. Arginine auxotrophy in ADPKD induces cell hyperproliferation, blocking the normal differentiation of renal tube cells and causing cyst formation. We explored the L-arginine (Arg)–nitric oxide (NO) molecular pathway in ADPKD, a multisystemic arginine auxotrophe disease. We developed a prospective case–control study that included a group of 62 ADPKD subjects with an estimated filtration rate over 60 mL/min/1.73 mp, 26 subjects with chronic kidney disease with an eGFR > 60 mL/min/1.73 mp, and a group of 37 healthy subjects. The laboratory determinations were the serum level of arginine, the enzymatic activity of arginase 2 and inducible nitric oxide synthase, the serum levels of the stable metabolites of nitric oxide (nitrate, direct nitrite, and total nitrite), and the endogenous inhibitors of nitric oxide synthesis (asymmetric dimethylarginine and symmetric dimethylarginine). In the ADPKD group, the levels of the arginine and nitric oxide metabolites were low, while the levels of the metabolization enzymes were higher compared to the control group. Statistical analysis of the correlations showed a positive association between the serum levels of Arg and the eGFR and a negative association between Arg and albuminuria. ADPKD is a metabolic kidney disease that is auxotrophic for arginine. Exploring arginine reprogramming and L-Arg–NO pathways could be an important element in the understanding of the pathogenesis and progression of ADPKD.

## 1. Introduction

The intrinsic dependence of a biological system on extracellular L-arginine is known as arginine auxotrophy [1]. Arginine auxotrophic cells are not capable of recycling or synthetizing intracellular arginine through the urea cycle [2,3]. Arginine auxotrophy is characteristic of a variety of cells. Arginine is limited in the rapid turnover of immune cells and tumoral cells [4,5,6,7,8,9]. This metabolic vulnerability has been documented in cellular proliferation, sepsis, inflammation, immunological tolerance, immunosuppression, carcinogenesis, cyst formation, and carcinogenesis (cutaneous melanoma, squamous cell carcinoma), solid cancers (prostate, liver, pancreas, lung, pleural mesothelioma, head and neck, clear cell renal carcinoma), and blood cancers (acute myeloid leukemia, lymphomas) [4,6,7,8,9,10,11,12,13,14,15,16,17,18,19].

Cells’ dependence on arginine could be influenced by different factors, such as the following: cell type, gene expression that encodes enzymes responsible for arginine metabolism, the cellular microenvironment, gene mutations, chromosomal structural changes, Th1/Th2 immunological polarization, spatial and temporal heterogeneity, metabolic plasticity, disease progression, and interconnection with other metabolic and inflammatory pathways [1,4,6,8,10,17,20,21,22,23,24].

The relationship between arginine auxotrophy and cyst formation in autosomal polycystic disease (ADPKD) was recently documented [6,25,26,27,28,29,30,31]. Currently, ADPKD is studied as a metabolic disease characterized by arginine reprogramming inducing abnormal cell proliferation and cyst formation [6,8,10,17,20,21,22,23,24,25,32].

ADPKD is the most frequently occurring hereditary cystic renal disease and affects over 13 million people all over the world, with an estimated incidence of 1:400 to 1:1000 births [33,34,35]. The main mutations detected in ADPKD are PKD1 (85–95%) on the 16p13 chromosome, PKD2 (10–15%) on the 4q21 chromosome, and PKD3 (2–3%) on the 11q12 chromosome. PKD genes codify polycystins (PC1 and PC2) localized in primary cysts, cell junctions, and extracellular matrices and ensure protein–protein and protein–carbohydrate interactions [33,34,35]. Experimental studies on animals and observational studies on humans suggest that cystogenesis is associated with a disorder of polycystic genes, endothelial lesions, intracellular calcium distribution, the activation of different cellular signaling pathways (cAMP, tyrosine kinase, Wnt, and mTOR), oxidant and antioxidant capacity disruption, ADMA accumulation, arginine auxotrophy, NO bio-availability, collagen deposits in interstitial space, mesangial cell hypertrophy, ischemia, and fibrosis, all leading to a decrease in renal function [6,33,34,35,36].

Cystogenesis and the phenotypic characteristics of ADPKD are not fully known. Cystogenesis is a focal process that initially affects 1–2% of nephrons [27]. ADPKD is a systemic, progressive disease characterized by cyst formation in the kidneys and other organs (liver, spleen, pancreas, and brain), renal function reduction, and large volume of the kidneys [25,28,29,30]. Metabolomic analysis of kidney cell culture with a deficit/excess of arginine showed that cystogenesis is dependent on arginine [6]. The authors of this paper aimed to analyze the L-arginine–nitric oxide molecular pathway in renal physiology and autosomal dominant polycystic kidney disease, and the results could offer new perspectives on the mechanism of cyst formation in ADPKD.

## 2. Materials and Methods

### 2.1. Study Participants

The present study is a prospective case–control study developed between 2021 and 2023, with patients selected from the Carol Davila Clinical Hospital of Nephrology and Victor Babes Clinical Hospital, Bucharest, Romania. The study protocol was approved by the Ethics Committee of the Carol Davila Clinical Hospital of Nephrology (10/11.04.2021). This study included a group of 62 patients with ADPKD, 26 subjects with chronic kidney disease (CKD) with an eGFR > 60 mL/min/1.73 mp, and 37 healthy subjects similar in sex and mean age.

The inclusion criteria in the study were as follows: patients over 18 years old with adequate nutritional status, and healthy subjects in the control group. The diagnosis of ADPKD was based on familial history, a clinical exam, and a CT/MRI scan. All patients in the ADPKD group had an eGFR > 60 mL/min/1.73 mp and no pathogenic treatment. The etiology of CKD was vascular nephropathy and tubulo-interstitial diseases (chronic pyelonephritis, lithiasis). The exclusion criteria were a history of arterial hypertension, cysts in organs other than the kidneys, an eGFR < 60 mL/min/1.73 mp, other renal disorders (history of hematuria, cysts infection, urinary tract infection, renal lithiasis), and metabolic disorders (carbamoyl phosphate synthase 1 and N-acetyl glutamate synthase deficiencies, lack of ornithine transcarbamilase, hyperargininemia, phenylketonuria). The characteristics of the groups are presented in Table 1.

### 2.2. Laboratory Determinations

Blood samples were collected from all study participants, after 12 h of fasting, using a holder–vacutainer system. The blood was kept for one hour at room temperature, and then centrifugated (3000× *g*, 10 min). The sera were separated and frozen at −80 degrees before analysis. Hemolyzed, icteric, lactescent, or microbiologically contaminated samples were excluded.

The serum L-Arg was assessed using an L-arginine assay kit (Sigma-Aldrich, catalog number MAK370, Darmstadt, Germany), which is an enzymatic test that converts L-Arg into intermediate molecules and develops a stable, colorimetric signal at 450 nm (A450). The absorbance was evaluated using the Tecan reader. The kit is simple to use, sensitive and specific.

The serum inducible nitric oxide synthase (NOS2, iNOS) activity was assessed using a colorimetric kit (CUSABIO Technology, catalog number CSB-E08148h, Houston, TX, USA) by the sandwich ELISA method. Its detection domain is 0.9–60 UI/mL, with a sensitivity of 225 UI/mL. The colorimetric determination of absorbance was carried out using the Tecan analyzer.

The serum ARG2 (L-amidine-hydrolase arginine; L-urea-hydrolase arginine; kidney arginase) was assessed using the Human Arginase II kit (catalog number MBS705280, Cambridge UK) by the sandwich ELISA method. Its detection domain is 0.47 UI/mL–39 U/L, with a sensitivity of 0.12 U/L. In the first step, the specific antibody for ARG2 interacted with the standard and patient serum. The second step consisted of adding a biotin-conjugated antibody, specific for ARG2, and then horseradish peroxidase conjugated with avidine. The substrate developed color proportionally with the initial ARG2 quantity, and the absorbance was measured at 450 nm by the Tecan analyzer (Tecan Global, Männedorf, Switzerland).

For the direct and total nitrite, the nitrate assessment was based on the enzymatic conversion of nitrate to nitrite by nitrate reductase, followed by the colorimetric detection of nitrite with an azo dye product, which absorbed light at 540–570 nm. The absorbance was measured by the Tecan analyzer, using the Cayman Chemical kit (CAY 780001, Cayman Chemical, Ann Arbor, MI, USA).

The symmetric and asymmetric dimethylarginines 2 were assessed by the ELISA method’s competitive variant, a sensitive, specific, and reproductible method, with no cross-reactions or interference with other structural analogs. The technique uses one unspecific antigen and a second one specific to the primary antibody. The absorbance was measured at 450 nm, which is inversely proportional to the substrate concentrations. The determinations were made using a human SDMA and ADMA ELISA kit (Abcam ab213973, Cambridge, UK).

### 2.3. Statistical Analysis

The data were analyzed using IBM SPSS Statistics 2015. The results are presented as the mean and standard deviation. Data comparison was performed using either ANOVA, with Tukey’s post hoc test or the Kruskal–Wallis test for normally distributed data, or, for non-normally distributed data, using Dunn’s post hoc test. Pearson’s correlation coefficient was used for evaluating the relationships among parameters. The Kolmogorov–Smirnov test was used to evaluate the data normality, before the assessment. For testing the hypothesis, 0.05 (5%) was the chosen level of significance, with a 95% confidence interval.

## 3. Results

### 3.1. Arginine and Its Metabolites in ADPKD

In the ADPKD patients, the serum levels of arginine had statistically significant lower levels, with a decrease of 1.87-fold compared to the control group (Table 2). In the CKD group, the arginine decreased 1.38-fold compared to the control group (Table 2).

The levels of the principal enzymes that metabolize arginine—arginase 2 and inducible nitric oxide synthase—were lower, while the endogenous inhibitors of nitric oxide synthesis were higher in the ADPKD group compared to the control group (Table 3). ARG2 decreased 1.03-fold, while the ARG2/Arg ratio increased 1.83-fold in the ADPKD group compared to the control group (*p* > 0.05). In the CKD group, ARG2 decreased 1.01-fold, while the ARG2/Arg ratio increased 1.62-fold compared to the control group (*p* > 0.05). There were no statistical differences regarding the ARG2 and ARG2/Arg variations when comparing the ADPKD and CKD groups (*p* > 0.05). iNOS decreased 1.22-fold (*p* < 0.01), while iNOS/Arg increased 1.57-fold (*p* < 0.001) in ADPKD compared to the control, and iNOS decreased 1.12-fold (*p* < 0.05) and NOS2/Arg increased 1.22-fold (*p* < 0.05) compared to the CKD group. In the CKD group, iNOS decreased 1.09-fold (*p* = 00.5), while NOS2/Arg increased 1.26-fold (*p* < 0.05) compared to the control group.

ADMA increased 1.51-fold (*p* < 0.02), while ADMA/Arg increased 3-fold (*p* < 0.001) in ADPKD compared to the control, and ADMA increased 1.31-fold (*p* < 0.05), while ADMA/Arg increased 1.8-fold (*p* < 0.05) compared to the CKD group. ADMA increased 1.15-fold (*p* < 0.05) and ADMA/Arg increased 1.66-fold when comparing the CKD and control groups. SDMA increased 2.65-fold (*p* < 0.01), and SDMA/Arg decreased 2-fold (*p* < 0.001) in ADPKD compared to the control, while SDMA increased 1.43-fold (*p* < 0.05) SDMA/Arg decreased 1.33-fold (*p* > 0.05) in ADPKD compared to the CKD group. When comparing the CKD and control groups, we detected 1.84-fold higher SDMA (*p* < 0.05) and 1.5-fold lower SDMA/Arg (*p* < 0.05) in the CKD group.

### 3.2. Nitric Oxide and Its Metabolites in ADPKD

The levels of the principal metabolites of NO were significantly lower in ADPKD compared to the control group (Table 4). Direct nitrite decreased 1.5-fold (*p* < 0.01), while direct nitrite/Arg increased 1.23-fold (*p* < 0.001) in the ADPKD group compared to the control and decreased 1.45-fold (*p* < 0.05) and 1.06-fold (*p* > 0.05), respectively, compared to the CKD group. In the CKD group, we detected a decrease of 1.05-fold (*p* > 0.05) of direct nitrite and an increase of 1.29-fold (*p* < 0.05) compared to the control group.

The total nitrite decreased 1.44-fold (*p* < 0.01), while the total nitrite/Arg increased 1.28-fold (*p* < 0.001) in ADPKD compared to the control group, and they decreased 1.26-fold (*p* < 0.05) and increased 1.08-fold (*p* < 0.05), respectively, compared to the CKD group. In the CKD group, when compared to the control, we detected a decrease of 1.14-fold (*p* < 0.05) in the total nitrite and an increase of 1.18-fold in the total nitrite/Arg (*p* > 0.05).

The nitrate decreased 1.38-fold (*p* < 0.01), while the nitrate/Arg increased 1.4-fold (*p* < 0.001) in ADPKD compared to the control group. Compared to the CKD group, the nitrate decreased 1.13-fold (*p* < 0.01), while the nitrate/Arg increased 1.21-fold (*p* < 0.01). In the CKD group, the nitrate decreased 1.22-fold (*p* < 0.05), while the nitrate/Arg increased 1.15-fold (*p* < 0.05) compared to the control.

### 3.3. L-Arginine–Nitric Oxide Molecular Pathway and Biological Characteristics of ADPKD

The relationship between the components of the L-arginine–nitric oxide molecular pathway and the biological characteristics of ADPKD subjects were analyzed (Table 5). When assessing the relationships among the studied markers, we detected strong negative correlations among Arg, ARG2, NOS2, SDMA and DMA, and a positive significant correlation between Arg and the total nitrite and nitrate. ARG2 correlated negatively with NOS2 and the total nitrite and nitrate and positively with SDMA and ADMA. NOS2 correlated positively with the total nitrite, nitrate, SDMA, and ADMA. The total nitrite correlated significantly negatively with SDMA and ADMA.

Interestingly, a negative relationship was detected between BMI and Arg, ARG2, and the total nitrite, and a positive one between BMI and NOS2. CRP and IL-6 negatively correlated with Arg. Urinary beta 2-microglobulin and UACR significantly correlated positively with ARG2. The eGFR had a strong statistically significant positive correlation with arginine and a negative one with ARG2 and NOS 2 in the ADPKD patients. No correlations were detected between the eGFR and the total nitrite, SDMA, or ADMA. Moreover, creatinine correlated with none of the studied markers.

## 4. Discussion

The kidneys are organs that express the genes responsible for L-arginine synthesis, conversion, and degradation, being mostly involved in L-arginine turnover. The present study showed the relationship between ADPKD and the serum deficiency of L-arginine. Normal levels of this amino acid are associated with a slow progression of ADPKD. The quantitative and time-course determination of L-Arg could help elucidate the physiopathology of ADPKD. Further experimental and observational studies could confirm and complete arginine auxotrophy in ADPKD [6,27].

**Figure 1:**
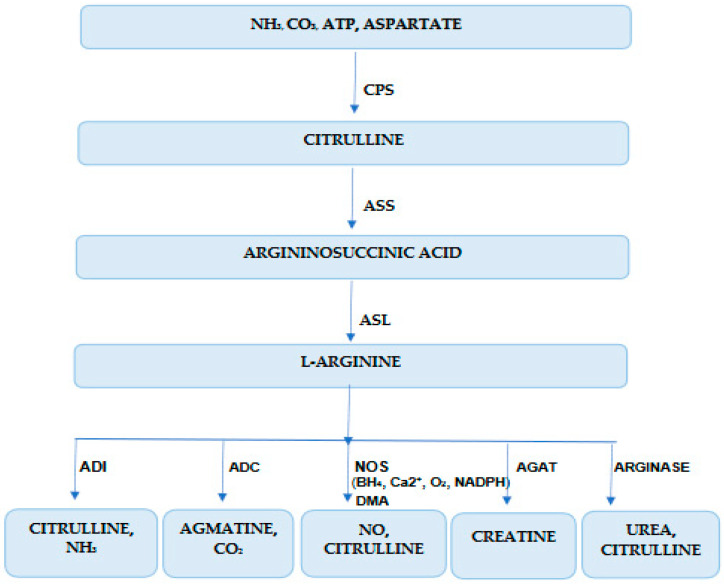
Arginine biochemistry in kidney disease. CPS (E.C. 6.3.4.16)—carbamoyl phosphate synthetase; ASS (E.C. 6.3.4.5)—arginine-succinate synthetase; ASL—arginine-succinate lyase (E.C. 4.3.2.1); ADI (E.C. 3.5.3.6.)—arginine deiminase, ADC (E.C. 4.1.1.19)—arginine decarboxylase, NOS (E.C. 1.14.13.39)—nitric oxide synthase, AGAT (E.C. 2.1.4.1.)—arginine:glycine amidino-transferase; NADPH—nicotinamide adenine dinucleotide phosphate, BH4—6R-5,6,7,8-tetrahydrobiopterin; DMA—dimethylarginine.

Knowing the renal metabolism of L-Arg could help us more easily understand the results of the present study. L-Arg production (2-amino-5-guanidin-valerian acid) in the human organism comes from: diet (5%), de novo biosynthesis from intracellular metabolites (15%), and import from the extracellular environment (80%) [8,36,37] (Figure 1). Arginine auxotrophy is determined by three cellular mechanisms:

- Sub-regulation of the enzyme that recycles arginine from precursors;
- Altered capacity of arginine transportation in cells;
- Overregulation of the enzymes that catabolize arginine.

Cells’ capacity to synthetize arginine from intracellular precursors by urea-cycle enzymes is altered in arginine-dependent cells. Enzymes involved in the trans-cellular synthesis of arginine are arginine succinate synthetase 1 (ASS1, E.C. 6.3.4.5), arginine succinate liaise (ASL E.C. 4.3.2.1), and ornithine trans-carbamylase (OTC E.C. 2.1.3.3) (Figure 1). ASS1 generates arginine-succinate from citrulline and aspartate and limits the endogen production of arginine. It is suppressed in murine and human ADPKD. ASL, which catalyzes arginine-succinate degradation in Arg and fumaric acid, is altered in arginine auxotrophic cells (Figure 1). OTC, which catalyzes ornithine conversion in carbamoyl phosphate and citrulline in mitochondria, is very important in arginine recirculation [6,36,37,38,39,40,41,42] (Figure 1).

Due to accelerated metabolism in ADPKD, the hyperproliferative cells need high concentrations of arginine. Arginine is transported from the extracellular microenvironment in the cytoplasm by cationic amino-acid transporters (SLC7A1, SLC7A2, SLC7A3, SLC7A4, SLC3A2) [17]. Arginine is degraded by arginase 1 (ARG1) and arginase 2 (ARG2) in ornithine and urea; inducible nitric oxide synthase (NOSi, E.C. 3.1.14.1.39) in NO and citrulline; arginine deaminase (ADI, E.C. 3.5.3.6) in citrulline and ammoniac; arginine decarboxylase (ADC, E.C. 4.1.1.19) in agmatine and carbon dioxide; arginine:glycine amidine -transferase (AG:AT, E.C. 2.1.4.1.) in creatine [5,43,44]. The final step in this pathway is catalyzed by ARG2 (an enzyme situated in the renal cortex and proximal tubes) and allows the urea to be used for excretion and ornithine regeneration for this cycle [2,17].

The present study shows that ADPKD is associated with the serum deficiency of L-arginine. CKD is also associated with low levels of L-arginine, but the variations were weak compared to ADPKD. The statistical analysis of the correlations showed a positive association between the serum levels of Arg and the eGFR, and a negative association between Arg and albuminuria. However, the positive and evolutive determination of L-Arg in ADPKD could be useful in the management of disease progression. An evaluation of the L-Arg–NO molecular pathway in renal physiology and ADPKD could offer more information about arginine auxotrophy, based on experimental studies on animals and observational analysis in humans [6,27]. The global reduction of arginine is a major component of phenotypic changes in ADPKD. The low levels of L-Arg that we found in ADPKD could be partially explained by ASS1 and ASL suppression, NOS2 and ARGG2 competition for the substrate, and an overproduction of ADMA and SDMA. Clear cell renal cell carcinoma and ADPKD were studied as metabolic arginine auxotrophe disorders [2,19,25,31,32,45,46]. This metabolic vulnerability has both similarities and differences when comparing the two discussed renal pathologies. The link between the biosynthetic pathways of arginine and glutamine by citrulline, the metabolomic analysis of kidney cells lines developed with a deficit/excess of arginine, and the depletion/supplementation of L-Arg through diet or pharmacological intervention showed compensatory, dose-dependent changes in arginine expression, ASS1 serum levels, cell proliferation, and cysts formation [6,39,47,48,49]. In ADPKD cells, L-Arg is important for membrane component synthesis, permitting a rapid increase in and expansion of cysts. In malignancies, L-Arg promotes tumor cell proliferation and immune alteration of malignant cells. However, L-Arg acts as a cyst formation modulator, while in ccRCC, it acts as an onco- and immune nutrient [6,10,47]. Arginine has a bidirectional effect on cell proliferation [6,47,48,49].

A recent study documented the prospective relationship between the initial concentrations of serum arginine and cancer risk [49]. Higher levels of arginine showed an increased risk of general or digestive cancer development [49]. Another recent study that assessed the relationship between the serum levels of L-Arg and cyst formation in children with ADPKD showed that arginine, ornithine, and putrescine, the urea and nitric oxide cycles, asparagine and glutamine metabolism, the methylation cycle, and the quinurenine pathway were significantly modified in ADPKD [48].

Glutamine dependence in ADPKD, investigated by the ASS1 pathway, with urea production in mice and human ADPKD tissue, showed that arginine depletion induced cyst reduction [6]. L-Arg deficiency detected in the present study is an important element for NOS and ARG function and activities, ADMA/SDMA production, and low production of NO (nitrites/nitrates). These results confirm the hypothesis of the mutual inhibition of the L-Arg metabolization pathways by NOS and ARG. L-Arg and NO bioavailability are reduced, NOS2 and ARG2 are overexpressed, the global production of nitrites/nitrates is low, and the serum levels of ADMA and SDMA are increased before the reduction in the eGFR in ADPKD patients. All these events are accentuated with ADPKD progression.

Recent studies have shown that the involvement of NOS and ARG in ADPKD progression is linked with: renal production of L-Arg, L-Arg metabolization by different pathways, renal alteration of protein activity/stability/expression, DDHA inactivation and increase in ADMA levels, reduction in renal NO production, and biphasic regulation of NO. NOS contribution is associated with the proliferative function of NOS2-positive macrophages, endothelial function, and oxidative stress [41,50,51,52,53]. The ARG2 contribution to cytogenesis in ADPKD is linked with ammonia detoxification in the urea cycle, the production of ornithine used for proline and polyamines synthesis, a decrease in NO synthesis by substrate limitation, a reduction in disponible arginine by affecting the MAPK and mTOR, and apoptosis induction [53,54,55].

The interaction between the two concurrent pathways of L-Arg metabolization was recently studied. NOS affects ARGs by substrate competition, high levels of endogenous inhibitors of ARG2, especially Nω-hydroxy-l-arginine (an intermediate metabolite of arginine conversion in NO), and nitrite accumulation [50,52,54,55]. ARG2 inhibits NOS by substrate limitation and high levels of endogenous inhibitors of NOS, especially ADMA and SDMA [27]. The mechanisms of cross-inhibition are responsible for the remodeling of the renal metabolism of L-Arg. However, NO inhibits ODC by enzyme S-nitrosylation, agmatine inhibits ODC and suppresses polyamine synthesis, putrescine and polyamines transport, while agmatine aldehyde inhibits NOS2 [53,54,55,56].

A deficiency of L-arginine, an increase in endogenous NOS inhibitors, a decrease in the activity of NOS, and increased levels of asymmetric dimethyl arginine (ADMA), a potent endogenous competitive inhibitor of NOS, in CKD were documented over time in some studies [57,58]. The results of our study confirm L-arginine deficiency in CKD. In ADPKD, we detected an important alteration of the L-Arg–NO pathway, which was more accentuated than in CKD.

In conclusion, L-Arg plays the role of an enzymatic substrate and regulatory molecule in signal transduction pathways in cells [53,56]. According to the results of the present study, the metabolic base of arginine auxtrophy in ADPKD is complex. The L-Arg–NO molecular pathway in renal cystogenesis versus renal physiology is disrupted in early stages of the disease and progresses with ADPKD. The relationship between arginine auxotrophy and the organ phenotypic variability of ADPKD requires complex genetic, metabolic, cellular, and clinical approaches.

Investigation into the transport and signaling mechanisms of arginine [56] and identifying the genetic and metabolic bases of arginine auxotrophy [59], the physio-pathological role of arginine derivates, and the interaction or competition between the enzymes involved in arginine metabolism [60,61,62] could represent important elements for understanding the pathogenesis and progression of ADPKD. The present study provides new data regarding the role of L-arginine–nitric oxide molecular pathway disturbances in ADPKD. Our study followed patients with ADPKD with an eGFR over 60 mL/min/1.73 m^2^. Further studies with a larger number of patients with an eGFR lower than 60 mL/min/1.73 m^2^ should be developed. For better descriptions of the roles of these factors in ADPKD physiopathology, a future study should follow patients for a longer period of time and collect data according to disease evolution. Arginine has many functions in human physiology and pathology. Many disorders mediated by arginine and its metabolites have been intensively studied recently [63,64,65,66,67,68,69]. The equilibrium of arginine production in the human body by physiological adaption or by pharmacological therapies could be an efficient method for the prevention or treatment of L-arginine–NO pathway metabolic alteration-associated diseases [63,64,65,66,67,68,69].

## 5. Conclusions

The metabolic phenotype of renal ADPKD cells was defined by alteration of the L-arginine-NO molecular pathway, a significant reduction in systemic arginine, increased activities of the principal enzymes that metabolize arginine, and reduction of the synthesis and bioactivity of NO. However, ADPKD could be considered a metabolic kidney disease, auxotrophic for arginine. L-Arg plays the role of enzymatic substrate and regulatory molecule in signal transduction pathways in ADPKD cells, which are the metabolic basis of arginine auxtrophy in ADPKD, being complex. Determining the relationship between arginine auxotrophy and organ phenotypic variability in ADPKD requires complex genetic, metabolic, cellular, and clinical approaches.

## Figures and Tables

**Table 1:** Patient characteristics.

	ADPKD (62 Subjects)	CKD (26 Subjects)	Control (37 Subjects)	*p* Value
Patient characteristics				
Female/male	29/33	15/21	17/20	p1 = 0.07
				p2 = 0.12
				p3 = 0.19
Age (years)	41.3 ± 10.1	42.1 ± 12.6	40.8 ± 9.2	p1 = 0.26
				p2 = 0.34
				p3 = 0.09
BMI (kg/m^2^)	23.1 ± 2.1	22.9 ± 3.2	22.3 ± 2.9	p1 = 0.71
				p2 = 0.56
				p3 = 0.21
Systolic blood pressure (mmHg)	12.2 ± 1.8	12.5 ± 1.4	11.8 ± 2.2	p1 = 0.08
				p2 = 0.06
				p3 = 0.13
Diastolic blood pressure (mmHg)	6.2 ± 0.9	6.5 ± 0.7	6.4 ± 0.7	p1 = 0.12
				p2 = 0.37
				p3 = 0.21
Laboratory data				
Hemoglobin (g/L)	12.9 ± 1.9	12.6 ± 2.1	13.7 ± 0.9	p1 = 0.09
				p2 = 0.11
				p3 = 0.07
Leukocytes (cells/mmc)	5492.50 ± 1035.7	5673 ± 1293	4997.3 ± 567.9	p1 = 0.05
				p2 = 0.09
				p3 = 0.16
Creatinine (mg/dL)	1.14 ± 0.39	1.21 ± 0.42	0.79 ± 0.19	p1 = 0.01
				p2 = 0.24
				p3 = 0.04
Albumin (g/dL)	3.9 ± 0.9	4.0 ± 1.2	4.2 ± 0.6	p1 = 0.07
				p2 = 0.09
				p3 = 0.52
CRP (mg/dL)	2.8 ± 1.7	2.3 ± 2.0	0.9 ± 0.9	p1 = 0.01
				p2 = 0.21
				p3 = 0.04
IL-6 (ng/dL)	9.5 ± 5.3	8.4 ± 4.7	4.2 ± 1.6	p1 = 0.03
				p2 = 0.36
				p3 = 0.04
HDL cholesterol (mg/dL)	39.2 ± 15.3	36.0 ± 18.3	50.5 ± 10.3	p1 = 0.04
				p2 = 0.09
				p3 = 0.04
LDL cholesterol (mg/dL)	147.4 ± 25.1	153.2 ± 33.6	109.7 ± 11.3	p1 = 0.03
				p2 = 0.11
				p3 = 0.05
Hematuria (SW cells/camp)	10.5 ± 8.3	14.0 ± 12.1	5 ± 5	p1 = 0.04
				p2 = 0.02
				p3 = 0.02
Leukocyturia (SW cells/camp)	12.1 ± 10.7	14.8 ± 13.5	3.5 ± 2.3	p1 = 0.01
				p2 = 0.06
				p3 = 0.04
Urinary b2-microglobulin (mg/L)	0.23 ± 0.09	0.28 ± 0.11	0.10 ± 0.04	p1 = 0.02
				p2 = 0.04
				p3 = 0.01
UACR (mg/g creatinine)	19.9 ± 5.1	24.6 ± 7.8	7.3 ± 0.4	p1 = 0.02
				p2 = 0.18
				p3 = 0.01
eGFR (mL/min/1.73 m^2^)	78.9 ± 15.1	74.3 ± 13.8	104.9 ± 10.3	p1 = 0.01
				p2 = 0.07
				p3 = −0.01

CKD—chronic kidney disease; BMI—body mass index; CRP—C-reactive protein; IL-6—interleukin 6; UACR—urinary albumin-to-creatinine ratio; eGFR—estimated glomerular filtration rate; *p*—statistical significance (p1—ADPKD group versus control; p2—ADPKD group versus CKD group; p3—CKD group versus control).

**Table 2:** Serum ARG levels in ADPKD and control groups.

Metabolite	ADPKD (62 Subjects)	CKD (26 Subjects)	Control (37 Subjects)	*p* Value
Arg (µmols/L)	47.6 ± 16.8	64.6 ± 17.9	89.2 ± 12.4	p1 < 0.001
				p2 < 0.05
				p3 < 0.05

ADPKD—autosomal dominant polycystic kidney disease; Arg—arginine; *p*—statistical significance (p1—ADPKD group versus control; p2—ADPKD group versus CKD group; p3—CKD group versus control).

**Table 3:** Arginine metabolites in ADPKD and control groups.

Metabolites	ADPKD (62 Subjects)	CKD (26 Subjects)	Control (37 Subjects)	*p* Value
ARG2 (U/L)	7.9 ± 2.8	8.1 ± 3.3	8.2 ± 2.5	p1 > 0.05
				p2 > 0.05
				p3 > 0.05
ARG2/Arg	0.165 ± 0.032	0.125 ± 0.041	0.095 ± 0.014	p1 > 0.05
				p2 > 0.05
				p3 > 0.05
NOS2 (U/L)	10.5 ± 2.9	11.8 ± 3.6	12.9 ± 1.4	p1 < 0.01
				p2 < 0.05
				p3 = 0.05
NOS2/Arg	0.220 ± 0.049	0.182 ± 0.52	0.144 ± 0.026	p1 < 0.001
				p2 < 0.05
				p3 < 0.05
ADMA (µmols/L)	0.88 ± 0.33	0.67 ± 0.38	0.58 ± 0.04	p1 < 0.02
				p2 < 0.05
				p3 < 0.05
ADMA/Arg	0.018 ± 0.003	0.010 ± 0.004	0.006 ± 0.001	p1 < 0.001
				p2 < 0.05
				p3 < 0.05
SDMA (µmols/L)	1.38 ± 0.36	0.96 ± 0.32	0.52 ± 0.08	p1 < 0.01
				p2 < 0.05
				p3 < 0.05
SDMA/Arg	0.003 ± 0.001	0.004 ± 0.001	0.006 ± 0.001	p1 < 0.001
				p2 > 0.05
				p3 < 0.05

ADPKD—autosomal dominant polycystic kidney disease; Arg—arginine; ARG2—arginase 2 (E.C. 3.5.3.1); NOS2—inducible nitric oxide synthase (E.C. 4.1.1.19); ADMA—asymmetric dimethylarginine; SDMA—symmetric dimethylarginine; *p*-level of significance. (p1—ADPKD group versus control; p2—ADPKD group versus CKD group; p3—CKD group versus control).

**Table 4:** Nitric metabolites in ADPKD and control groups.

Metabolite	ADPKD (62 Subjects)	CKD (26 Subjects)	Control (37 Subjects)	*p* Value
Direct nitrite (µmols/L)	10.1 ± 2.2	14.5 ± 3.2	15.3 ± 2.9	p1 < 0.01
				p2 < 0.05
				p3 > 0.0.5
Direct nitrite/Arg	0.212 ± 0.035	0.224 ± 0.041	0.171 ± 0.024	p1 < 0.001
				p2 > 0.05
				p3 < 0.05
Total nitrite (µmols/L)	23.5 ± 6.6	29.7 ± 5.3	33.9 ± 3.6	p1 < 0.01
				p2 < 0.05
				p3 < 0.05
Total nitrite/Arg	0.493 ± 0.11	0.451 ± 0.09	0.380 ± 0.07	p1 < 0.01
				p2 > 0.05
				p3 < 0.05
Nitrate (µmols/L)	13.4 ± 7.8	15.2 ± 4.1	18.6 ± 4.4	p1 < 0.01
				p2 < 0.01
				p3 < 0.01
Nitrate/Arg	0.281 ± 0.07	0.23 ± 0.05	0.208 ± 0.03	p1 < 0.01
				p2 < 0.05
				p3 < 0.05

ADPKD—autosomal dominant polycystic kidney disease; Arg—arginine; *p*—level of significance (p1—ADPKD group versus control; p2—ADPKD group versus CKD group; p3—CKD group versus control).

**Table 5:** Relationships among L-arginine–nitric oxide markers and biological characteristics in ADPKD.

Parameters		Arg	ARG2	NOS2	Total Nitrite	SDMA	ADMA
ARG2	R	−0.43	-				
	*p*	<0.01	-				
NOS2	R	−0.86	−0.58	-			
	*p*	<0.001	<0.001	-			
Total nitrite	R	0.54	−0.23	0.39	-		
	*p*	<0.001	0.01	<0.01	-		
Nitrate	R	0.79	−0.38	0.46			
	*p*	<0.001	0.02	<0.001			
SDMA	R	−0.28	0.29	0.36	−0.49		
	*p*	<0.001	0.45	<0.01	<0.001		
ADMA	R	−0.80	0.15	0.49	−0.66		
	*p*	0 < 0.01	0.23	<0.001	0.001		
BMI	R	−0.40	−0.17	0.42	−0.35	0.03	0.26
	*p*	<0.01	0.38	0.01	<0.01	0.79	0.32
Hb	R	0.18	0.42	0.14	−0.11	0.04	0.14
	*p*	0.09	0.20	0.41	0.40	0.73	0.39
Leukocytes	R	0.13	0.19	0.14	0.01	0.04	0.12
	*p*	0.42	0.23	0.71	0.95	0.71	0.58
Creatinine	R	−0.31	−0.24	−0.11	0.12	0.04	0.15
	*p*	0.10	0.32	0.21	0.96	0.73	0.54
Albumin	R	0.17	−0.08	−0.34	0.07	−0.03	−0.18
	*p*	0.10	0.55	0.08	0.60	0.95	0.84
CRP	R	−0.27	0.19	0.26	−0.25	0.34	0.19
	*p*	0.011	0.16	0.15	0.08	0.89	0.12
IL-6	R	−0.37	0.29	0.20	−0.26	0.24	0.31
	*p*	0.012	0.15	0.25	0.13	0.09	0.34
HDL cholesterol	R	0.25	−0.18	−0.32	0.21	−0.08	−0.21
	*p*	0.070	0.11	0.43	0.38	0.76	0.22
LDL cholesterol	R	−0.41	0.27	0.28	0.18	0.01	0.26
	*p*	0.030	0.28	0.13	0.20	0.90	0.43
Hematuria	R	−0.14	0.09	0.08	−0.28	0.04	0.06
	*p*	0.31	0.65	0.73	0.09	0.95	0.54
Leukocyturia	R	−0.15	0.18	0.10	0.23	0.12	0.09
	*p*	0.22	0.43	0.48	0.54	0.75	0.55
Urinary b2-microglobulin	R	−0.26	0.35	0.32	0.08	0.26	0.33
	*p*	0.32	0.04	0.09	0.82	0.65	0.23
UACR	R	−0.24	0.41	0.12	−0.25	0.12	0.21
	*p*	0.07	0.03	0.74	0.06	0.12	0.28
eGFR	R	0.48	−0.27	−0.19	0.27	−0.21	−0.19
	*p*	<0.01	0.04	0.05	0.37	0.04	0.01

ADPKD—autosomal dominant polycystic kidney disease; Arg—arginine; ARG2—arginase 2 (E.C. 3.5.3.1); NOS2—inducible nitric oxide synthase (E.C. 4.1.1.19); ADMA—asymmetric dimethylarginine; SDMA—symmetric dimethylarginine; BMI—body mass index; CRP—C-reactive protein; HDL—high-density lipoprotein; LDL—low-density lipoprotein; UACR—urine albumin–creatinine ratio; eGFR—estimated glomerular filtration rate; R—coefficient correlation; *p*—level of significance.

## Data Availability

All research data are presented in the manuscript.

## Abbreviations

ADPKD	Autosomal dominant polycystic kidney disease
PKD	Polycystic kidney disease
CPS (E.C. 6.3.4.16)	Carbamoyl-phosphate synthetase
ASS (E.C. 6.3.4.5)	Arginine-succinate synthetase (E.C. 6.3.4.5)
ASL (E.C. 4.3.2.1)	Arginine-succinate lyase
ADI (E.C. 3.5.3.6.)	Arginine deiminase
ADC (E.C. 4.1.1.19)	Arginine decarboxylase
NOS (E.C. 1.14.13.39)	Nitric oxide synthase
AGAT (E.C. 2.1.4.1.)	Arginine:glycine amidinotransferase
ARG2 (E.C. 5.3.1)	Arginase (E.C. 3.5.3.1)
OTC (E.C. 2.1.3.3)	Ornithine transcarbamylase
NO	Nitric oxide
NADPH	Nicotinamide adenine dinucleotide phosphate
BH4	6R-5,6,7,8-tetrahydrobiopterin
PC1 and PC2	Polycystins
BMI	Body mass index
HDL	High-density lipoprotein
LDL	Low-density lipoprotein
CRP	C-reactive protein
IL-6	Interleukin 6
UACR	Urinary albumin-to-creatinine ratio
eGFR	Estimated glomerular filtration rate
L-Arg	Arginine
ADMA	Asymmetric dimethylarginine
SDMA	Symmetric dimethylarginine
